# Comparison of Diagnostic Accuracies of Various Endoscopic Examination Techniques for Evaluating the Invasion Depth of Colorectal Tumors

**DOI:** 10.1155/2012/621512

**Published:** 2012-10-04

**Authors:** Satomi Haruki, Kiyonori Kobayashi, Kaoru Yokoyama, Miwa Sada, Wasaburo Koizumi

**Affiliations:** Department of Gastroenterology, Kitasato University School of Medicine, Kanagawa, Sagamihara 252-0380, Japan

## Abstract

This study was designed to assess the clinical value of magnifying endoscopy combined with EUS for estimating the invasion depth of colorectal tumors. We studied 168 colorectal adenomas and carcinomas that were sequentially examined by conventional endoscopy followed by magnifying endoscopy and EUS in the same session to evaluate invasion depth. Endoscopic images obtained by each technique were reassessed by 3 endoscopists to determine whether endoscopic resection (adenoma, mucosal cancer, or submucosal cancer with slight invasion) or colectomy (submucosal cancer with massive invasion or advanced cancer) was indicated. The accuracy of differential diagnosis was compared among the examination techniques. The rate of correct differential diagnosis according to endoscopic examination technique was similar. The proportion of lesions that were difficult to diagnose was significantly higher for EUS (15.5%) than for conventional endoscopy and magnifying endoscopy. Among lesions that could be diagnosed, the rate of correct differential diagnosis was the highest for EUS (89.4%), but did not significantly differ among three endoscopic examination techniques. When it is difficult to evaluate the invasion depth of colorectal tumors on conventional endoscopy alone, the combined use of different examination techniques such as EUS may enhance diagnostic accuracy in some lesions.

## 1. Introduction 

Endoscopic examinations have an important role in the differential diagnosis of benign and malignant colorectal tumors (adenomas and carcinomas), as well as in accurate estimation of the depth of invasion and selection of the treatment. In particular, “early” colorectal cancer with invasion confined to the mucosa or the submucosa is a borderline lesion for the selection of either endoscopic resection or colectomy. It is thus essential to accurately evaluate the depth of tumor invasion on the basis of endoscopic findings. 

The invasion depth of colorectal cancer is basically estimated on conventional endoscopy combined with chromoendoscopy, as needed. If the depth of tumor invasion is difficult to estimate on conventional endoscopy alone, however, additional examinations such as magnifying endoscopy to assess pit patterns and endoscopic ultrasonography (EUS) are performed. However, few studies have examined the extent to which these detailed examination techniques improve the accuracy of estimating the depth of invasion. We studied colorectal tumors that were sequentially examined by conventional endoscopy followed by EUS and magnifying endoscopy in the same session to evaluate invasion depth. The accuracy of evaluating the depth of tumor invasion was then compared among these endoscopic examination techniques to determine whether the additional use of in-depth procedures improved the accuracy of estimating the depth of tumor invasion, thereby facilitating the selection of treatment. 

## 2. Methods

### 2.1. Patients

From January 2002 through April 2007, we sequentially examined 168 colorectal tumors (166 patients) by conventional endoscopy, followed by magnifying endoscopy to evaluate the pit pattern, and EUS to estimate the depth of tumor invasion at the request of the patients' attending physicians. After endoscopic resection or surgical operation, the invasion depth of all lesions was determined histopathologically. Lesions that were difficult to assess on conventional endoscopy because of factors such as inadequate bowel preparation and high-grade intestinal peristalsis were excluded. 

The invasion depth of the colorectal tumors was classified according to the Japanese classification of colorectal carcinoma, issued by the Japanese Society for Cancer of the Colon and Rectum [1]. There were 44 adenomas, 66 carcinomas with invasion confined to the mucosa (mucosal cancer), and 54 carcinomas with invasion of the submucosa (submucosal cancer) (Table 1). Among submucosal cancers, 15 lesions had a submucosal invasion depth of less than 1000 *μ*m (slight invasion), and 39 had an invasion depth of 1000 *μ*m or greater (massive invasion). The most common lesion location was the rectum (74 lesions), followed by the sigmoid colon, transverse colon, and ascending colon. The macroscopic type of the tumors was classified according to the Paris endoscopic classification [2] and system reported by Kudo et al. [3]. Laterally spreading tumors (LST) were most common (131 lesions, 78%) and included 57 granular-type LST and 74 nongranular-type LST. About half of all tumors (82 lesions, 49%) had a diameter of 20 mm or greater, and the mean tumor diameter was 24.1 ± 14.2 mm. As for treatment, 55 lesions were treated by endoscopic resection, 85 by colectomy, and 28 by transanal local resection or transanal endoscopic microsurgery. Colectomy was additionally performed to treat 2 lesions with massive submucosal invasion that initially underwent endoscopic resection. 

### 2.2. Colonoscopic Examination

For bowel preparation before colonoscopy, oral intestinal lavage (polyethylene glycol) was mainly performed. As premedication, scopolamine butylbromide (10 mg) or glucagon (1 mg) was given intramuscularly to suppress intestinal peristalsis. We used colonoscopy with magnifying function (PCF-Q240ZI or CF-2TQ240ZI, Olympus, Tokyo, Japan). All colonoscopic examinations, including magnifying endoscopy and EUS, were performed by a single endoscopist who had at least 20 years of experience in colonoscopy. The number of years of experience in detailed evaluations was 17 for EUS and 7 for magnifying endoscopy. During conventional endoscopy, most lesions were also examined by chromoendoscopy, performed by spraying the mucosa with 0.2% indigo carmine dye. Before colonoscopy, patients were given a detailed explanation of the examination objectives, methods, and possible complications. Written-informed consent for colonoscopic examination was obtained from all patients. 

### 2.3. Methods for EUS

After the completion of conventional endoscopy, the intestine near the tumor was filled with deaerated water that had been warmed to about body temperature, and EUS was performed to evaluate the depth of invasion. An ultrasound probe with a frequency of 20 MHz (UM-3R; Olympus, Tokyo, Japan) or a 3-dimensional ultrasound probe with a frequency of 20 MHz (UM DP20-25R; Olympus, Tokyo, Japan) was used. 

On EUS, the normal wall of the colon is basically visualized as a 5-layer structure. From the luminal side, the hyperechoic first layer and hypoechoic second layer correspond to the mucosa, the hyperechoic third layer to the submucosa, the hypoechoic fourth layer to the muscularis propria, and the hyperechoic fifth layer to the subserosa or serosa (adventitia) [4]. The invasion depth of the colorectal carcinomas and adenomas on EUS was evaluated to be the deepest layer that showed narrowing or rupture of the wall structure due to the tumor. The resolution of currently available EUS devices precludes adequate visualization of the thin muscularis mucosae of the colonic wall and accurate measurement of the depth of submucosal invasion by carcinomas [5]. Submucosal carcinomas were classified into two subgroups on the basis of the degree of submucosal invasion on EUS. If the superior margin of the third layer was slightly narrowed by the tumor, submucosal cancer with slight invasion was diagnosed. If the third layer was severely narrowed or ruptured, but the fourth layer remained intact, submucosal cancer with massive invasion was diagnosed. 

### 2.4. Magnifying Endoscopy

After the completion of conventional endoscopy and EUS, magnifying colonoscopic examination was performed to evaluate pit patterns. The tumor was washed with water to remove any mucus, sprayed with 0.2% indigo carmine dye, and examined by magnifying endoscopy at a magnification of 80 to 100 times to evaluate pit patterns. If pit patterns could not be accurately evaluated on indigo carmine staining alone, 0.05% crystal violet stain was concurrently applied. Pit patterns of the colorectal tumors were evaluated according to Kudo's classification [6]. Type V_I_ pit patterns were further classified as mildly irregular or severely irregular on the basis of structural and arrangement irregularities of pits, pit density, and stromal staining between pits. On the basis of the results of previous studies examining the relation between pit patterns and tumor invasion depth [6–10], tumors with type IIIs, III_L_, IV, or V_I_ mildly irregular pit patterns were considered to be indicated for endoscopic resection (adenoma, mucosal cancer, and submucosal cancer with slight invasion). Tumors with type V_I_ severely irregular or type V_N_ pit patterns were considered to be indicated for colectomy (submucosal cancer with massive invasion and advanced cancer). 

### 2.5. Evaluation of Invasion Depth

Three endoscopists who had no information on the histopathological findings of tumors reassessed the invasion depth of the colorectal tumors. All 3 endoscopists had at least 10 years of experience in colonoscopy and at least 5 years of experience in EUS and magnifying endoscopy. The number of years of experience did not differ appreciably according to examination technique among the 3 endoscopists. Conventional endoscopic, magnifying endoscopic, and EUS images were reviewed for lesions presented in random order. The lesions were divided into 2 groups on the basis of the estimated depth of invasion: lesions for which endoscopic resection was indicated (adenoma, mucosal cancer, and submucosal cancer with slight invasion) and those for which colectomy was indicated (submucosal cancer with massive invasion and advanced cancer). If the evaluation made by each endoscopist was consistent with the histopathological diagnosis for the resected specimen, the endoscopic diagnosis was classified as a correct diagnosis. If the evaluation did not agree with the histopathological diagnosis, the endoscopic diagnosis was classified as a misdiagnosis. If the invasion depth was difficult to evaluate on the basis of the presented endoscopic images, the endoscopic diagnosis was classified as a misdiagnosis. If at least 2 endoscopists made the same diagnosis, that diagnosis was considered the final diagnosis for the lesion. The accuracy of differential diagnosis and the frequency of difficult-to-diagnose lesions were retrospectively compared among conventional endoscopy, magnifying endoscopy, and EUS. For lesions that were considered by all 3 endoscopists to have an assessable invasion depth, the diagnostic accuracy was compared among the examination techniques. Our institutional review board approved the study protocol.

### 2.6. Statistical Analysis

Numerical data are expressed as means ± standard deviation. The chi-square test and Fisher's exact test were used to compare frequencies among groups. *P* values of less than 0.05 were considered to indicate statistical significance. StatView software (version 5.0 for Windows, SAS Institute Inc., Cary, NC) was used for statistical analysis.

## 3. Results

### 3.1. Accuracy of Differential Diagnosis according to Examination Technique

The rate of correctly diagnosing lesions for which endoscopic resection was indicated (i.e., adenoma, mucosal cancer, and submucosal cancer with slight invasion) was 83.2% (104/125 lesions) on conventional endoscopy, 83.2% (104/125) on magnifying endoscopy, and 81.6% (102/125) on EUS. The rate of correctly diagnosing lesions for which colectomy was indicated (i.e., submucosal cancer with massive invasion and advanced cancer) was 76.7% (33/43 lesions) on conventional endoscopy, 79.1% (34/43) on magnifying endoscopy, and 79.1% (34/43) on EUS. The overall accuracy of differential diagnosis was similar for conventional endoscopy (81.5%), magnifying endoscopy (82.1%), and EUS (81.0%) (Table 2). 

### 3.2. Frequency of Difficult-to-Diagnose Lesions

 The percentage of lesions that were evaluated by at least 1 of the 3 endoscopists to be difficult to diagnose on endoscopic images was 3.0% for conventional endoscopy, 4.8% for magnifying endoscopy, and 15.5% for EUS (Table 3). The frequency of difficult-to-diagnose lesions was significantly higher for EUS than for conventional endoscopy and magnifying endoscopy.

### 3.3. Comparison of Diagnostic Accuracy among Lesions Able to Be Diagnosed

The number of lesions for which the invasion depth was considered assessable by all 3 endoscopists was 163 for conventional endoscopy, 160 for magnifying endoscopy, and 142 for EUS. The rate of correct diagnosis among lesions with assessable endoscopic images was highest for EUS (89.4%), followed by magnifying endoscopy (85.6%) and conventional endoscopy (82.8%). The diagnostic accuracy of EUS was the highest, but did not significantly differ among three endoscopic examination techniques (Table 4). 

### 3.4. Lesions for Which Magnifying Endoscopy and EUS Were Useful for Diagnosis

**Figure 1:**
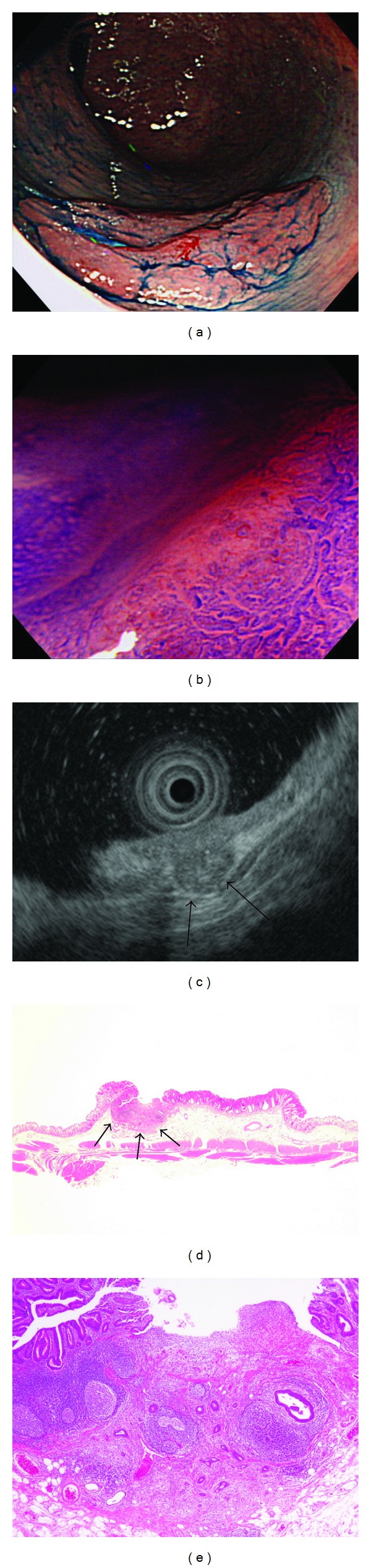
(a) Colonoscopic images after spraying with 0.2% indigo carmine dye, showing a nongranular LST in the rectum. The surface showed mild redness. Extensibility of the tumor on insufflation was relatively good. (b) Magnifying endoscopic images after the application of 0.05% crystal violet stain, showing a type V_N_ pit pattern characterized by an amorphous structure of part of the tumor. (c) EUS images, showing severe narrowing of the third layer in part of the tumor (arrow). Cancer with deep submucosal invasion was diagnosed. (d) and (e) Histopathological findings of the surgically resected specimen. (d) The longest diameter of the tumor was 20 mm. Although most of the tumor was confined to the mucosa, part of the lesion invaded the middle layer of the submucosa (arrow). The diagnosis was a well-differentiated tubular adenocarcinoma. (e) The intramucosal part of the tumor had become detached at the site of submucosal invasion.

Endoscopic examination of a nongranular-type LST after spraying the tumor with 0.2% indigo carmine dye showed that the extensibility of the tumor on insufflation was relatively good, and all 3 endoscopists considered endoscopic resection to be indicated for treatment (Figure 1(a)). However, magnifying endoscopy after the application of 0.05% crystal violet stain showed pits with an amorphous structure (type V_N_ pit pattern) in part of the tumor (Figure 1(b)). On EUS, the third layer of the wall was severely narrowed in part of the tumor (Figure 1(c)). On the basis of the magnifying endoscopic and EUS findings, all 3 endoscopists judged that colectomy was indicated for treatment. Histopathological examination of the surgically resected specimen revealed a well-differentiated tubular adenocarcinoma invading the middle layer of the submucosa (Figures 1(d) and 1(e)). The vertical depth of invasion in the submucosa was 1850 *μ*m. There was no evidence of lymphovascular invasion or lymph-node metastasis. 

## 4. Discussion

 Among colorectal cancers, mucosal cancer can be resected endoscopically because there is no risk of metastasis. In particular, the recent development of techniques such as endoscopic submucosal dissection and endoscopic piecemeal mucosal resection has enabled the endoscopic resection of even large lesions [11–13]. Submucosal cancers have a risk of metastases to lymph nodes and other organs [14–17]. However, tumors with slight submucosal invasion depth of less than 1000 *μ*m are very rarely associated with metastasis; endoscopic resection is thus indicated for the treatment of such lesions [14]. In contrast, tumors with massive submucosal invasion of 1000 *μ*m or deeper carry a risk of metastasis and must therefore be treated by colectomy with lymph-node dissection. Early colorectal cancer should therefore be differentially diagnosed according to the depth of invasion as either mucosal cancer or submucosal cancer with slight invasion or as submucosal cancer with massive invasion. The most appropriate treatment method (endoscopic resection or colectomy) should then be selected. 

The invasion depth of colorectal cancer is generally evaluated on endoscopic examination. The basic procedure is conventional endoscopy. The depth of invasion is estimated on the basis of tumor diameter and macroscopic findings, as well as other characteristics of the tumor surface, such as a cracked or distended appearance, friability, spread, and fold convergence [18, 19]. As for the macroscopic findings, the frequency of submucosal cancer is higher among superficial-type tumors than elevated-type tumors [18]. Saitoh et al. [19] reported that a distended appearance, a deep depression, an uneven depressed surface, and convergent folds are important endoscopic findings that suggest a depressed-type, early colorectal cancer deeply invading the submucosa. They also reported that mucosal cancer or submucosal cancer with slight invasion could be differentiated from submucosal cancer with massive invasion on conventional endoscopy combined with chromoendoscopy for more than 90% of lesions. However, if the diagnosis is equivocal on conventional endoscopy, a number of additional examinations have been recommended, such as the evaluation of pit pattern of the tumor surface on magnifying endoscopy and the assessment of EUS findings, vascular patterns on narrow band imaging [20, 21], and non-lifting signs before endoscopic mucosal resection [22]. 

The evaluation of pit patterns on magnifying endoscopy is useful for differentiating neoplastic from nonneoplastic colorectal polyps [23, 24], as well as for estimating the invasion depth of early colorectal cancers [7–10]. Pit patterns of colorectal polyps on magnifying endoscopy are most often evaluated according to the classification of Kudo et al. [6]. Pit patterns of types I and II are associated with a high frequency of nonneoplastic lesions; type III_S_, III_L_, and IV with adenomatous polyps; and type V with cancer. Type V pit patterns can be further classified into type V_I_ and type V_N_. Intype V_N_, the pit structure has been lost and is amorphous, suggesting submucosal cancer with massive invasion [6]. Type V_I_ is subclassified into type V_I_ with mild irregularity and type V_I_ with severe irregularity on the basis of findings such as narrowed pit lumens, irregular margins,unclear outlines, and decreased or absence of stromal staining between pits [10]. The former suggests mucosal cancer or submucosal cancer with slight invasion, whereas the latter suggests submucosal cancer with massive invasion [7–10]. In our study, type V_I_ pit patterns were subdivided into type V_I_ with mild irregularity and type V_I_ with severe irregularity. The presented tumors were then reevaluated to decide whether endoscopic resection (adenoma, mucosal cancer, or submucosal cancer with slight submucosal invasion) or colectomy (submucosal cancer with massive invasion or advanced cancer) was indicated. 

Many studies have reported that EUS is useful for estimating the invasion depth of colorectal cancer [25–30]. In particular, the advent of ultrasound probes able to be inserted through the forceps channel of an endoscope has allowed lesions to be evaluated by EUS after conventional endoscopy, greatly simplifying the endoscopic procedure [25]. We previously studied the diagnostic usefulness of EUS with respect to the selection of treatment for early colorectal cancer. The rate of correctly differentiating mucosal cancer and submucosal cancer with slight invasion from submucosal cancer with massive invasion was 90%, indicating good diagnostic accuracy [26]. 

Pit patterns on magnifying endoscopy and EUS findings have been confirmed to be useful for evaluating the invasion depth of colorectal tumors. However, few studies have compared the diagnostic accuracy of conventional endoscopy, magnifying endoscopy, and EUS in large numbers of lesions. Some studies have reported that the diagnostic accuracy of EUS is superior to that of magnifying endoscopy [31, 32], whereas others have shown that the diagnostic accuracy is similar [33]. Consensus has thus not been reached. One prospective study comparing magnifying endoscopy with EUS in patients with submucosal cancer [32] showed that EUS has a significantly higher diagnostic accuracy than magnifying endoscopy. 

The present study compared the diagnostic accuracies of conventional endoscopy, magnifying endoscopy, and EUS by reviewing endoscopic images to estimate the invasion depth of mainly early colorectal tumors and thereby select the treatment method. Because the macroscopic appearance and disease stage of tumors can differ on endoscopic examinations performed at different times, we only studied lesions that were sequentially examined by conventional endoscopy, magnifying endoscopy, and EUS in the same session. Moreover, to ensure that invasion depth was objectively evaluated, 3 endoscopists who were blinded to the histopathological diagnosis of the tumors reviewed the endoscopic images.

When only lesions with interpretable endoscopic images as assessed by all 3 endoscopists were evaluated, the diagnostic accuracy of EUS was the highest, but did not differ from that of magnifying endoscopy and tended to be higher than that of conventional endoscopy (*P* = 0.0978). Among gastrointestinal endoscopic examinations, the depth of invasion is estimated on the basis of changes of the tumor surface on conventional endoscopy and magnifying endoscopy. In contrast, with EUS the entire lesion can be visualized in vertical slices, allowing the invasion depth to be objectively evaluated on the basis of changes in wall structure. 

However, the diagnosis of colorectal tumors on EUS has several limitations. Histologically, the presence of inflammation or fibrosis around the tumor invasion front may lead to overestimation of the depth of invasion [34]. In addition, clear ultrasonographic images are occasionally precluded by factors such as the macroscopic type and location of tumors. In our study, although examinations were performed by an endoscopist who had more than 15 years of experience in EUS of the colorectum, about 15% of lesions were difficult to diagnose on EUS, which was significantly higher than percentages of difficult-to-diagnose lesions on conventional endoscopy and magnifying endoscopy. Matsunaga et al. [35] reported that 12% of early colorectal cancers were difficult to clearly visualize on EUS. Colorectal tumors arising in the colonic flexure, on folds, or near the anus are often difficult to visualize. Inadequate filling of the colon with deaerated water caused by intestinal peristalsis may also adversely affect the visualization of tumors. We previously reported that many lesions difficult to visualize on EUS are located in the proximal colon, associated with marked haustral thickening and frequent intestinal peristalsis [26]. Devices and examination techniques for EUS should therefore be further refined. Even on magnifying endoscopy, an appreciable number of lesions were difficult to diagnose because of factors such as mucus adhering to the tumor surface or bleeding. 

This study compared the accuracy of estimating the invasion depth of mainly colorectal LST among 3 different endoscopic techniques. The invasion depth was correctly diagnosed on conventional endoscopy combined with chromoendoscopy for more than 80% of lesions. The relatively high diagnostic accuracy of conventional endoscopy may be attributed to the following factors: a high proportion of lesions were adenomas and mucosal cancers, for which it is relatively easy to estimate the invasion depth; the endoscopists who performed the examinations and estimated the invasion depth were well experienced. Because many conventional endoscopic findings used to evaluate invasion depth are subjective, diagnostic accuracy may largely depend on the knowledge and experience of the endoscopist. A previous study has reported that the accuracy of estimating the invasion depth of colorectal cancer on conventional endoscopy is negatively affected if the examination is performed by an inexperienced endoscopist [35].

Among in-depth evaluations of colorectal tumors, the assessment of pit patterns on magnifying endoscopy, especially the classification of type V_I_ pit patterns [10], is often difficult for inexperienced physicians. The most important endoscopic findings at the time of evaluation remain controversial among specialists. Another problem is the high proportion of difficult-to-diagnosis lesions, even on EUS. However, when the depth of tumor invasion is difficult to estimate on conventional endoscopy, the results of our study suggest that the concurrent use of in-depth examinations such as EUS may be useful for diagnosis in some lesions. In our study, the years of experience of the endoscopist who performed all colonoscopic examinations was longer for EUS than for magnifying endoscopy. Such differences in the number of years of experience may have influenced the diagnostic outcomes of these examination techniques. Further prospective multicenter studies may be needed to compare the diagnostic accuracies of various endoscopic techniques and to establish new strategies for the endoscopic diagnosis of colorectal cancer. 

## Figures and Tables

**Table 1:** Clinical characteristics of the study subjects.

(1) Histology	
Adenoma	44 (26%)
M ca^†^	66 (39%)
SM-S ca^‡^	15 (9%)
SM-M ca^¶^	39 (23%)
MP ca^#^ or over	4 (3%)
(2) Location	
Rectum	74 (44%)
Sigmoid	34 (20%)
Descending	10 (6%)
Transverse	21 (13%)
Ascending	20 (12%)
Cecum	9 (5%)
(3) Morphology	
Protruded	20 (12%)
Superficial	13 (8%)
LST* granular	57 (34%)
LST* nongranular	74 (44%)
Others	4 (2%)
(4) Size (mm)	
~9	14 (8%)
10~19	55 (33%)
20~	82 (49%)
unknown	17 (10%)

^†^M ca: mucosal cancer, ^‡^SM-S ca: submucosal slight invaded cancer.

^¶^SM-M ca: submucosal massive invaded cancer.

^#^MP ca: muscularis propria invaded cancer, *LST: laterally spreading tumor.

**Table 2:** Comparison of diagnostic accuracy among 3 different endoscopic techniques (conventional endoscopy, magnifying endoscopy, and EUS).

	Correct	Error	Accuracy
Conventional endoscopy	137	31	81.5%^A^
Magnifying endoscopy	138	30	82.1%^B^
EUS	136	32	81.0%^C^

*P* = 0.8875 (A versus B), *P* = 0.7785 (B versus C), *P* = 0.8888 (A versus C).

**Table 3:** Comparison of the frequencies of lesions with endoscopic images those were difficult to diagnosis among 3 different endoscopic techniques (conventional endoscopy, magnifying endoscopy, and EUS).

	Inadequate imaging		Frequency of inadequate
	Yes	No	imaging lesions
Conventional	5	163	3.0%^A^
endoscopy			
Magnifying	8	160	4.8%^B^
endoscopy			
EUS	26	142	15.5%^C^

*P* = 0.3961 (A versus B), *P* = 0.0011 (B versus C), *P* < 0.0001 (A versus C).

**Table 4:** Comparison of diagnostic accuracy among 3 different endoscopic techniques after excluding lesions with inadequate images.

	Correct	Error	Accuracy
Conventional endoscopy	135	28	82.8%^A^
Magnifying endoscopy	137	23	85.6%^B^
EUS	127	15	89.4%^C^

*P* = 0.4897 (A versus B), *P* = 0.3188 (B versus C), *P* = 0.0978 (A versus C).
